# National epidemiology of culture-confirmed brucellosis in Israel, 2004–2022

**DOI:** 10.1017/S0950268824000803

**Published:** 2024-06-26

**Authors:** Miriam Weinberger, Jacob Moran-Gilad, Michal Perry Markovich, Svetlana Bardenstein

**Affiliations:** 1 Faculty of Medicine, Tel Aviv University, Tel Aviv, Israel; 2 Department of Infectious Diseases, Shamir (Assaf Harofeh) Medical Center, Zerifin, Israel; 3 Department of Health Policy and Management, School of Public Health, Faculty of Health Sciences, Ben Gurion University of the Negev, Beer Sheva, Israel; 4 Israel Veterinary Services, Beit Dagan, Israel; 5 Department of Bacteriology, Kimron Veterinary Institute, Beit Dagan, Israel

**Keywords:** Brucella, brucellosis, brucellosis epidemiology, brucellosis incidence, *Brucella melitensis*, epidemiology, humans, Israel, incidence, zoonosis

## Abstract

Brucellosis, a global zoonosis, is endemic in Israel. We used a national database of culture-confirmed cases (2004–2022) to analyse the trends of brucellosis. Of 2,489 unique cases, 99.8% were bacteraemic, 64% involved males, and the mean age was 30.5 years. *Brucella melitensis* was the dominant species (99.6%). Most cases occurred among the Arab sector (84.9%) followed by the Jewish (8.5%) and Druze (5.5%) sectors. The average annual incidence rates overall and for the Arab, Druze, and Jewish sectors were 1.6/100,000, 6.6/100,000, 5.5/100,000, and 0.18/100,000, respectively. The annual incidence rates among the Arab (incidence rate ratio (IRR) = 36.4) and the Druze (IRR = 29.6) sectors were significantly higher than among the Jewish sector (p < 0.001). The highest incidence rates among the Arab sector occurred in the South District, peaking at 41.0/100,000 in 2012. The frequencies of *B. melitensis* isolated biotypes (biotype 1 – 69.1%, biotype 2 – 26.0%, and biotype 3 – 4.3%) differed from most Middle Eastern and European countries. A significant switch between the dominant biotypes was noted in the second half of the study period. Efforts for control and prevention should be sustained and guided by a One Health approach mindful of the differential trends and changing epidemiology.

## Introduction

Brucellosis is a worldwide zoonotic infection transmitted mainly by cattle, swine, goats, sheep, and dogs [1]. According to recent estimates, the annual global incidence of human brucellosis is significantly higher than previously assumed and amounts to 1.6–2.1 million new cases annually [2]. Humans acquire the infection through the consumption of infected animal products, mostly unpasteurized milk, and dairy products, by direct contact with the wool and skin of infected animals and their secretions (especially following abortions), or by inhalation of infected aerosols [2]. Another route of transmission is traditional slaughter rituals [3]. Human brucellosis is mainly caused by *Brucella melitensis* and less frequently by *Brucella abortus*, *Brucella suis*, and *Brucella canis.* Although seldom lethal, brucellosis is a chronic and debilitating infection in humans. In addition to its health burden in endemic countries, brucellosis impacts animal welfare and is associated with substantial economic loss to agriculture [2].

Control measures have been successful in reducing the incidence of brucellosis in most developed countries, but they still pose a heavy burden in many parts of the world, particularly Africa, Asia, and Central America. Some hot spots of increased incidence include the Middle East, the Mediterranean basin, the Balkans, and the Persian Gulf [2]. Europe is known to have the most advanced brucellosis surveillance and control programmes. Many European countries are considered brucellosis-free, reporting only travel-associated cases. A sharp decrease in the annual incidence rate was noted in the European Union (EU) countries from 0.1/100,000 population in 2007 [4] to 0.04/100,000 in 2022 with the highest incidence rate found in Greece (0.33/100,000) [5]. Outside the EU, several Balkan countries like Albania, Macedonia, Bosnia, and Herzegovina are known to be highly endemic [6, 7]. In the United States, brucellosis is rare, with less than 200 new cases reported annually [8].

Brucellosis is endemic in Israel, affecting mainly sheep and goats, dairy cattle, and humans [9]. Several outbreaks were also traced to infected camel milk [10]. Since the eradication of *B. abortus* in the mid-1980s, the only species responsible for infection in Israel has been *B. melitensis* [9]. The annual incidence rate in humans has declined from 6.0/100,000 in the late 1950s to roughly 2.0/100,000 thereafter. It increased again sharply in the mid-1980s and early 1990s, with a peak of 11.0/100,000. Following successful eradication campaigns, the incidence rates declined and remained low at 2.0/100,000 for two decades, only to rise again in 2013/4 [11]. Higher incidence rates were reported among the Arab sector (mainly of the Bedouin communities), who carry the heaviest burden of brucellosis in Israel, yet comprise circa one-fifth of the overall population [12, 13]. Large outbreaks were also reported among the Druze sector [14]. These trends in the Arab and Druze sectors are mainly attributed to small agriculture, particularly family-owned herds of sheep, goats, and sometimes camels, raised traditionally according to a mix of sedentary and pastoral farming systems. Individual consumption and door-to-door marketing of unpasteurized fresh soft dairy products are important underlying factors for the spread of brucellosis beyond herd-owning families [13].

Vaccination of sheep and goats by the ocular Rev 1 *B. melitensis* vaccine and cattle by the subcutaneous B19 vaccine is mandatory in Israel. However, challenges in the enforcement of these regulations among family-owned herds may have led to a high proportion of unvaccinated animals, thus becoming the major source of brucellosis among those communities [15–17].

Reporting of human brucellosis is mandatory by law in Israel. The surveillance of brucellosis is overseen by the Division of Epidemiology, Israel Ministry of Health (IMOH), and relies on a case definition that is based on a compatible clinical description, with either laboratory confirmation and/or epidemiological linkage to a laboratory-confirmed case. Between 1998 and 2009, only 64% of cases reported to the Division of Epidemiology were laboratory-confirmed, using serology or culture [13]. Moreover, it is estimated that under-reporting may be in the range of 50% [18].

We sought to study the national epidemiology of brucellosis in Israel based on culture-confirmed cases and characterize the trends of brucellosis with respect to the various ethnic groups (sectors) and *Brucella* biotypes.

## Methods

Human brucellosis in Israel has been a notifiable disease by law since 1951. All isolates of *Brucella* spp. from clinical laboratories countrywide are passively submitted to the National Reference Laboratory at the Kimron Veterinary Institute (KVI), Ministry of Agriculture and Rural Development, Beit Dagan, for confirmation, final identification to the species level, and biotyping. A culture-confirmed *Brucella* case was defined as a patient with a positive *Brucella* culture reported to KVI. Multiple positive cultures with the same *Brucella* biotype occurring in the same patients within 6 months were considered a single case of infection, since a certain proportion of patients may relapse or present with persistent bacteraemia. *Brucella* cases between the years 2004 and 2022 were analysed.

Demographic data (age, sex, sector, residence) of the infected patients were obtained from the KVI laboratory records. The city of residence was attributed to the six administrative districts of Israel to calculate incidence rates in each district according to the Israel Central Bureau of Statistics (ICBS) [19].

The overall number of *Brucella* cases reported to IMOH was obtained from weekly epidemiological reports [20]. Demographic data for the Israeli population were obtained from the annual Statistical Abstracts of Israel published by the ICBS for the entire country and for the six administrative districts of Israel (Jerusalem, North, Haifa, Center, Tel Aviv, and South) [21]. Population data for the year 2022 were extrapolated by multiplying the population size in 2021 by the per cent increase reported between 2020 and 2021.

### Statistical methods

Incidence rates were calculated by dividing the number of culture-confirmed cases by the population size for each study year and according to age, sex, sector, and the administrative district of residency. Average incidence rates were calculated by dividing the sum of annual incidence rates by the number of study years in the studied period.

The chi-square test was used to compare the rates of children less than 18 years between the different sectors.

To compare between incidence rates, we used quasi-Poisson regression models with the logarithm of the population size used as an offset. The quasi-Poisson regression models were used to account for overdispersion. Univariate quasi-Poisson regression models were used to compare the incidence rates of culture-confirmed cases between the various sectors (Jewish, Arab, and Druze), between the two *B. melitensis* major biotypes (biotype 1 and biotype 2) in the Arab sector, and between the six administrative districts in the Arab sector.

Multivariate quasi-Poisson regression models were compiled for each administrative district in the Arab sector to compare the average annual incidence rates of the two *B. melitensis* major biotypes adjusted for two time periods (2004–2012 and 2013–2022). The models were fitted to the data using the glm function in the R package stats using R software version 4.3.1 (R Foundation®).

A heat map and Israel district maps for visual presentation of the incidence rates were constructed using the R software version 4.3.1 (R Foundation®).

### Ethical considerations

The study was approved by the local ethical committee of the Shamir (Assaf Harofeh) Medical Center, Zerifin, Israel (#168/13). Data were de-identified, and only anonymized demographic data were used for analyses.

## Results

During the 19 study years (2004–2022), a total of 2,749 isolates of *Brucella* were submitted to KVI. Two hundred and sixty isolates were excluded, including 22 isolates from persons living outside Israel and 238 repeated isolates occurring within the first six months of the first isolation in the same person. A total of 2,489 culture-confirmed brucellosis cases were thus available for analysis. Of these, 25 (1.0%) represented a second case of infection in the same person. Over the course of the study years, a total of 4,864 brucellosis cases were reported to the IMOH (based on the national case definition) [13]. Therefore, culture-confirmed cases accounted for 52.2% of the nationally reported cases (annual average: 59.5%, range: 35.1%–91.3% annually).

**Figure 1.**
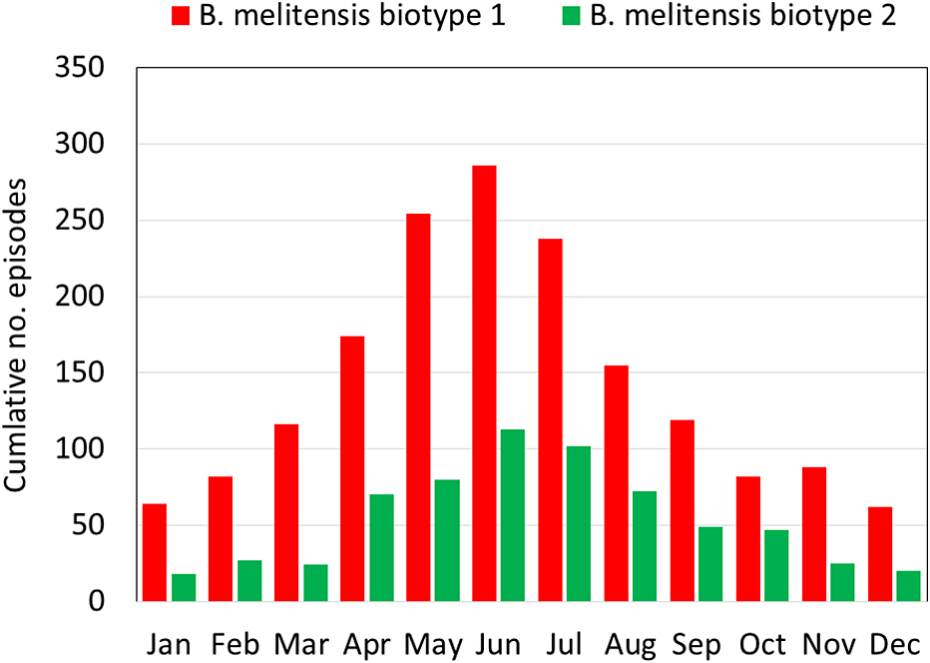
Seasonal pattern of culture-confirmed *Brucella* cases according to *Brucella melitensis* major biotypes, Israel, 2004–2022.

The source of isolation and the infecting species are depicted in Table 1. The vast majority (2,484, 99.8%) involved bloodstream infection, two involved the central nervous system and occurred in children aged one and 16 years, and two involved pregnancies. The dominant species was *B. melitensis* (99.6%) and the dominant biotype was *B. melitensis* biotype 1, which was responsible for 69.1% of the cases, followed by *B. melitensis* biotype 2 (26.0%) and *B. melitensis* biotype 3 (4.3%). Three cases of *B. abortus* occurred in persons who worked in dairy farms abroad (Turkey − 2 and Georgia − 1), and only one was reported from a local farm. Five cases were associated with brucellosis vaccination activities, implicating the Rev 1 *B. melitensis* vaccine strain. Across the study years, 72.3% of the cases occurred in the warm months between April and September. A seasonal pattern was evident for the two major biotypes – *B. melitensis* biotype 1 and *B. melitensis* biotype 2 (Figure 1).

**Table 1.** Source of *Brucella melitensis* isolation and patients’ sector according to biotype, Israel, 2004–2022, No. (%)

	*B. melitensis* biotype 1	*B. melitensis* biotype 2	*B. melitensis* biotype 3	*B. melitensis* spp.	Total^a^
No. of cases	1,720 (69.3)	647 (26.1)	106 (4.3)	7 (0.3)	2,480 (100)
Source of isolation^a^					
Blood	1,710 (69.3)	646 (26.2)	105 (4.3)	7 (0.3)	2,468 (100)
Blood and synovial fluid	4 (80)	1 (20)			5 (100)
Blood and uterine cavity			1 (100)		1 (100)
Synovial fluid	3 (100)				3 (100)
Blood and CSF	1 (100)				1 (100)
CSF	1 (1,000)				1 (100)
Aborted foetus	1 (100)				1 (100)
Sector					
Arab	1,450 (68.7)	557 (26.4)	97 (4.6)	6 (0.3)	2,110 (100)
Jew	148 (71.8)	51 (24.8)	6 (2.9)	1 (0.5)	206 (100)
Druze	104 (76.4)	30 (22.1)	2 (1.5)		136 (100)
Other/unknown	18 (64.3)	9 (32.1)	1 (3.6)		28 (100)

Abbreviation: CSF, cerebrospinal fluid.

a Nine additional cases were due to *Brucella abortus* (four cases, all from bacteraemic patients of the Jewish sector) and Rev 1 *B. Melitensis* vaccine strain (five cases, all from bacteraemic patients, three from the Arab sector and two from the Jewish sector).

The mean age of the patients was 30.5 years (median 26.0, interquartile range (IQR) 12.7–46.3, range 4 months–95 years). Males accounted for 1,546 (64.1%) of culture-confirmed brucellosis cases. Arab was the main sector affected, accounting for 84.9% of the cases, followed by the Jewish (8.5%), Druze (5.5%), and other/unknown (1.1%) sectors. Across the study period, the Arab sector accounted for 19.4–21.2% of the Israeli population and the Druze sector accounted for 1.6%, while the Jewish sector was the majority group accounting for 76.4–73.8% of the overall population.

**Figure 2.**
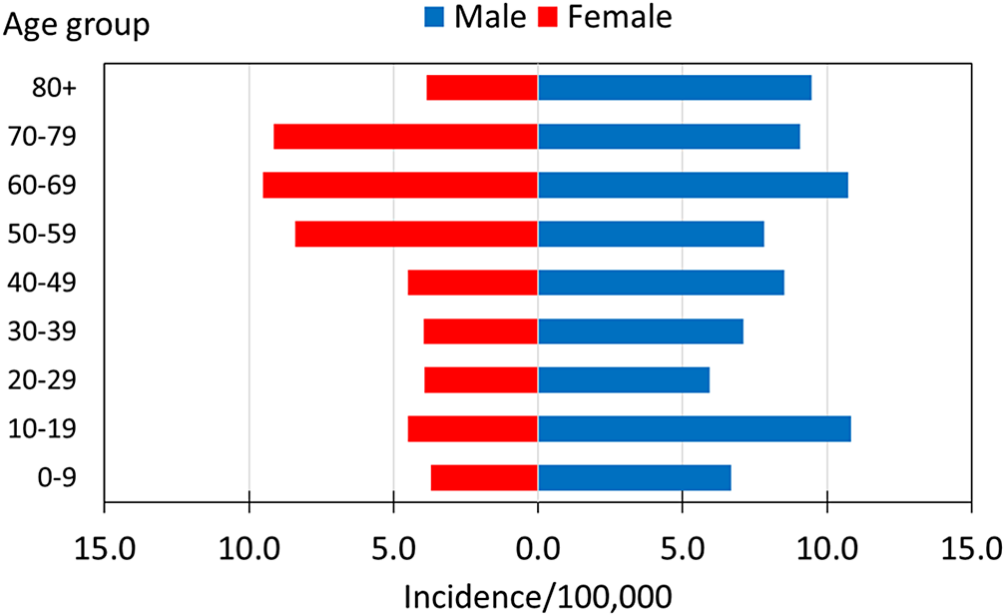
Average age-related culture-confirmed *Brucella* incidence rates among the Arab sector, Israel, 2004–2022.

The average age-related incidence is shown for the Arab sector (Figure 2). Incidence rates among males were roughly double for most age groups relative to females, except for the sixth to eighth decades of life, which showed a comparable incidence. Among males, the highest burden occurred both in the second decade of life and later in the sixth to eighth decades. Among females, the incidence rate increased towards the sixth decade of life. The percentage of children less than 18 years was highest among the Arab sector (42.3%) compared to the Druze (24.8%) and Jewish (11.6%) sectors (P < 0.001).

**Figure 3.**
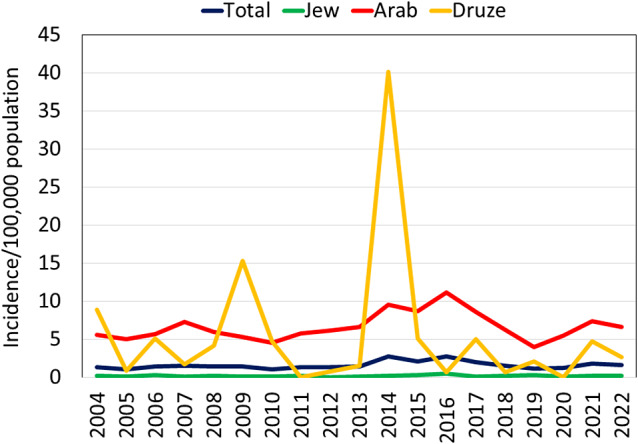
Annual incidence rates of culture-confirmed *Brucella* cases by sector, Israel, 2004–2022.

The annual incidence rates of culture-confirmed *Brucella* cases across the study years are presented in Figure 3. The overall national annual incidence rate increased gradually from 2004 to peak in 2014, reaching 2.9/100,00 population, followed by a decrease to 1.8/100,000 in the last study year. The overall average annual incidence rate across the study years was 1.6/100,000 and differed among the various sectors as follows: 0.18/100,000 among the Jewish sector, 5.5/100,000 among the Druze sector, and 6.6/100,000 among the Arab sector. The annual incidence rates among the Arab (incidence rate ratio (IRR) = 36.4, 95 per cent confidence interval (95% CI): 23.3–60.2) and Druze (IRR = 29.6, 95% CI: 14.1, 60.0) sectors were significantly higher than among the Jewish sector (p < 0.001).

The annual incidence trends for the Arab sector generally paralleled the overall national trends with a peak of 11.1/100,000 in 2016. Throughout the study years, the ratio of the annual brucellosis incidence rates of the Arab sector compared to the Jewish sector ranged from a low of 14.2 in 2019 to a high of 369.0 in 2012 and was 61.9 on average.

The annual incidence rates among the Druze sector demonstrated two sharp increases to 15.3/100,000 in 2009 and 40.2/100,000 in 2014. These peaks were 2.9-fold and 4.2-fold higher than the incidence rate among the Arab sector in the respective years. Two Druze settlements, Yirka and Yanuh-Jat, accounted for 67.7% (92/136) of the cases among the Druze sector, while the other cases were distributed among 15 other settlements. In 2009, the incidence rate in these two settlements was 27.0 and 107.1/100,000, respectively, and in 2014, it was 129.0 and 243.8/100,000, respectively.

**Figure 4.**
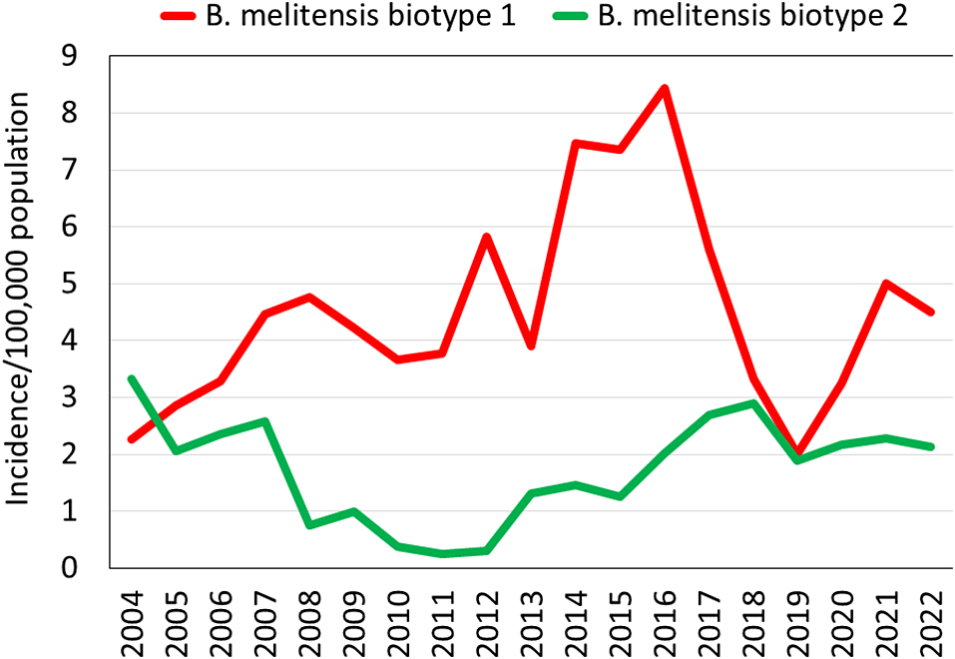
Annual incidence rates of culture-confirmed *Brucella* cases among the Arab sector according to the major *Brucella melitensis* biotypes, Israel, 2004–2022.

The distribution of brucellosis cases according to the various *B. melitensis* biotypes and per sector is shown in Table 1. Among the Arab sector, the annual incidence rates of *B. melitensis* biotype 1 were higher than those of *B. melitensis* biotype 2 across the study years (IRR = 2.60, 95% CI: 1.94–3.54, p < 0.0001) (Figure 4). It increased 3.7-fold from 2004 to 2016 and then showed a sharp decrease. The incidence of *B. melitensis* biotype 2 decreased 10.8-fold between 2004 and 2012 and then increased gradually to roughly two-thirds of the initial incidence in 2004. Among the Jewish and Druze sectors, the annual incidence trends of *B. melitensis* biotype 1 were similar to those of the overall annual trends encompassing all biotypes.

The average annual incidence rates of culture-confirmed *Brucella* cases according to the six Israeli administrative districts and per sector are shown in Table 2. The number of cases and the incidence rates among the Jewish sector were low in all districts. All brucellosis cases among the Druze sector were reported from the North and Haifa districts in northern Israel, where the Druze communities are located. In the North District, the high peak incidence rates were driven by the aforementioned outbreaks in 2009 and 2014 in the Druze settlements of Yirka and Yanuh-Jat.

**Table 2.** Distribution of culture-confirmed *Brucella* cases according to patients’ sector in six administrative districts, Israel, 2004–2022 (incidence/100,000)^a^

Israel administrative districts	Jerusalem	North	Haifa	Center	Tel Aviv	South
Arab sector						
No. of cases	286	536	189	98	10	991
Average incidence rate	4.4	4.0	4.1	3.1	2.4	24.3
Nadir/peak incidence rate	0/14.8	1.0/8.6	0.4/14.4	0/16.9	0/20.2	12.0/41.0
Jewish sector						
No. of cases	20	32	36	26	10	73
Average incidence rate	0.2	0.3	0.3	0.1	0.0	0.4
Nadir/peak incidence rate	0/0.6	0/1	0/1.1	0/0.5	0/0.2	0.1/1.1
Druze sector						
No. of cases	0	128	8	0	0	0
Average incidence rate	0	6.4	1.8	0	0	0
Nadir/peak incidence rate	0/0	0/49.8	0/8.6	0/0	0/0	0/0

a The addresses of 46 patients were unknown or outside these districts.

**Figure 5.**
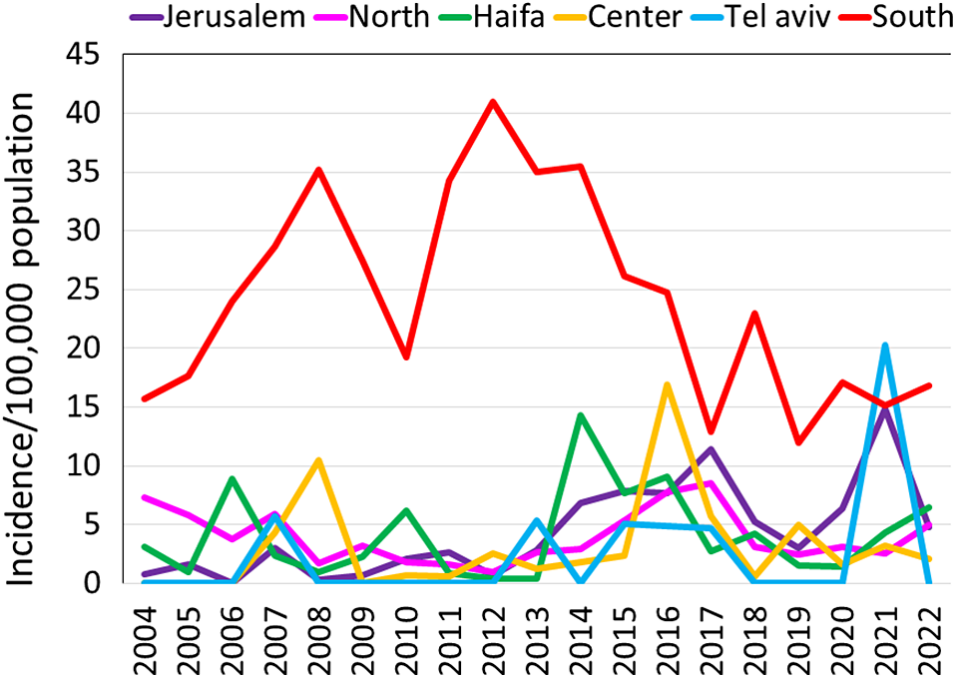
Annual incidence rates of culture-confirmed *Brucella* cases among the Arab sector according to the Israel administrative districts, Israel, 2004–2022.

The annual incidence rates for the Arab sector were further analysed (Figure 5). They were highest in the South District, where most of the Arab Bedouin communities reside. There was a highly significant difference between the South District and each of the five other districts (p < 0.0001) as follows: Jerusalem (IRR = 0.20, 95% CI: 0.14–0.29), North (IRR = 0.17, 95% CI: 0.12–0.22), Haifa (IRR = 0.18, 95% CI: 0.11–0.27), Center (IRR = 0.14, 95% CI: 0.07–0.23), and Tel Aviv (IRR = 0.11, 95% CI: 0.01–0.44). The annual incidence rates in the South District increased from 15.7 in 2004 to a peak of 41.0/100,000 in 2012 and then sharply decreased to reach 16.8/100,00 in 2022.

**Figure 6.**
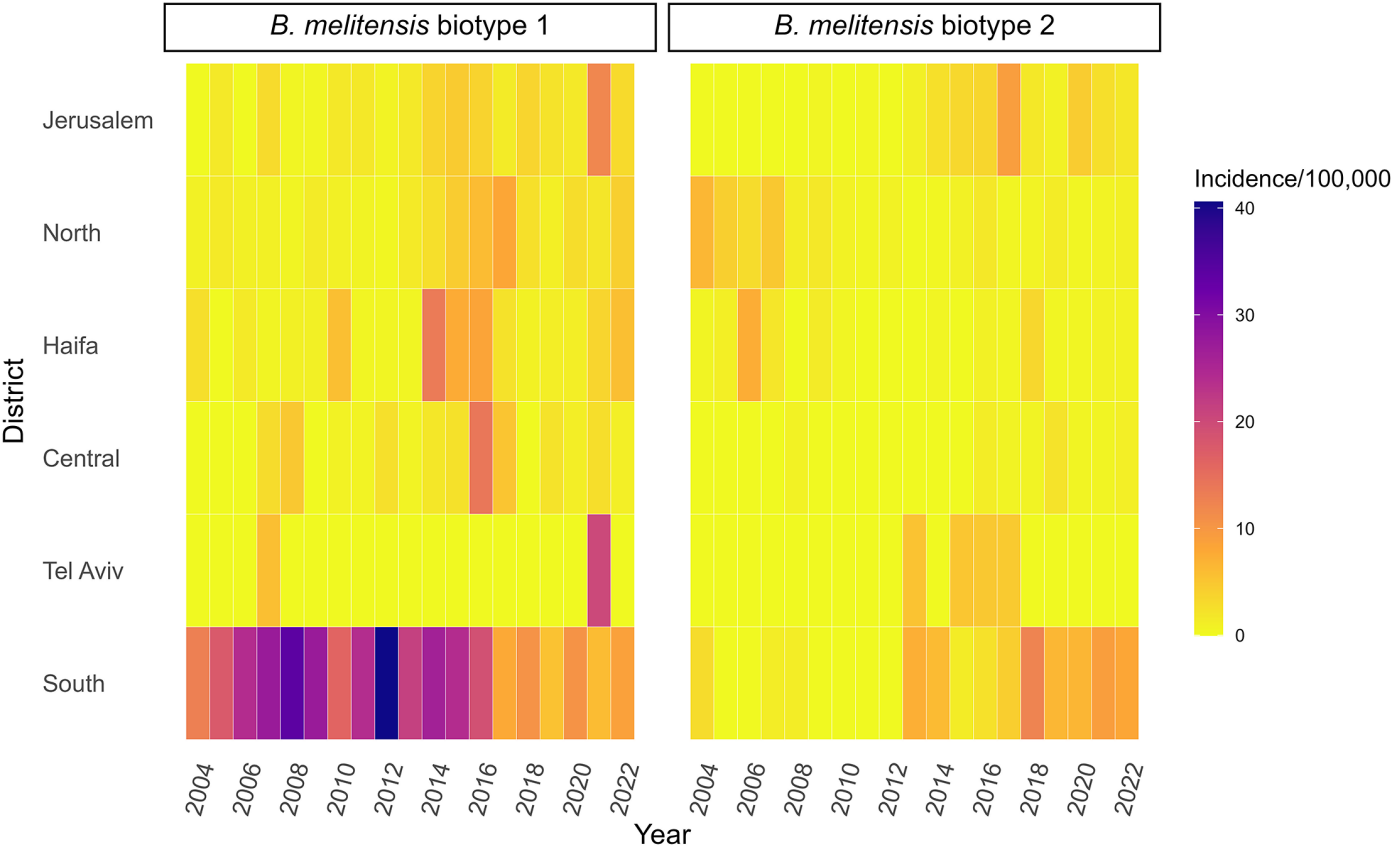
Heat map describing the annual incidence rates of *Brucella melitensis* biotypes 1 and 2 among the Arab sector according to the study years and the Israel administrative districts.

**Figure 7.**
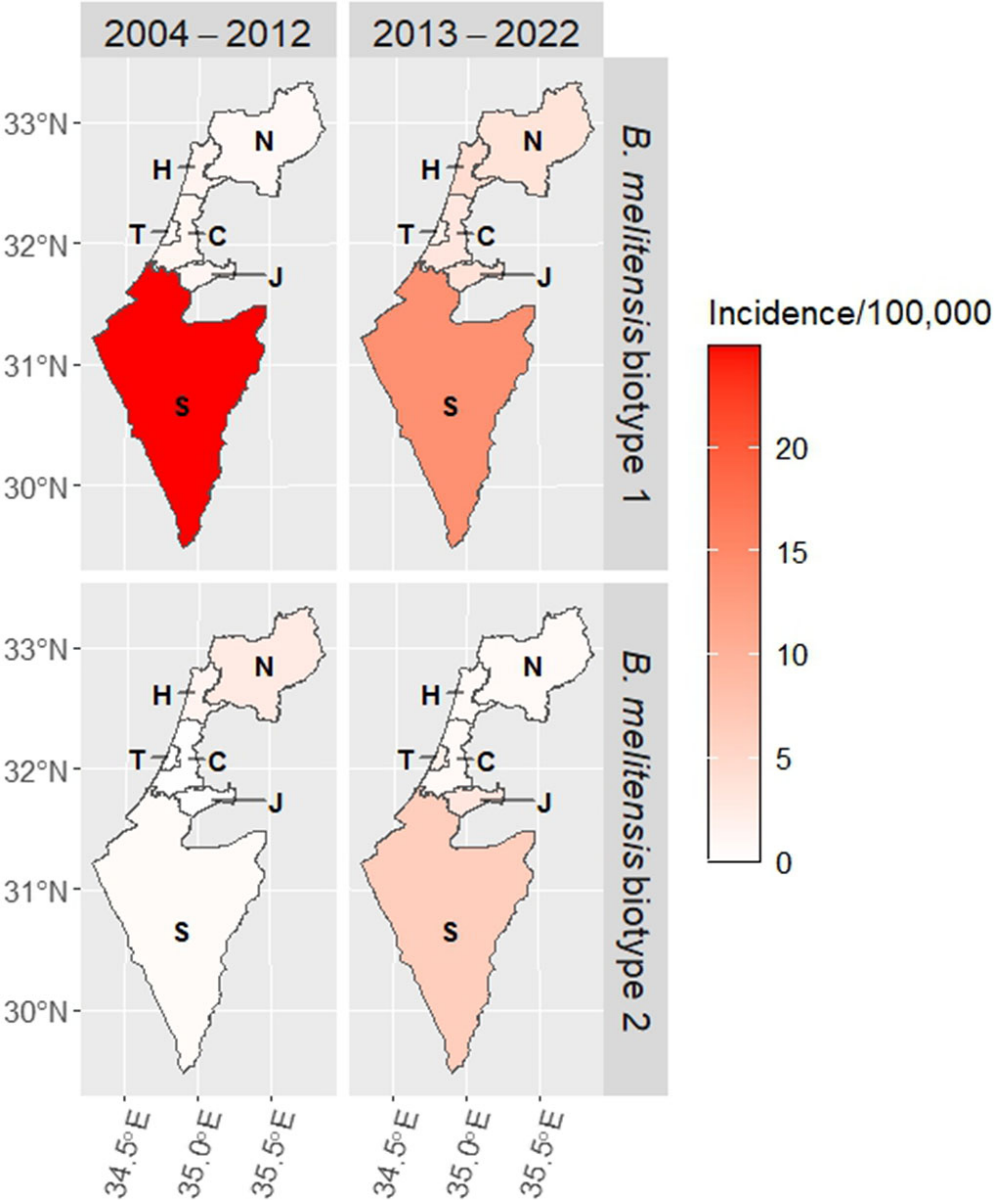
*Brucella melitensis* average incidence rates in the period 2004–2012 compared to the period 2013–2022 according to *B. melitensis* biotypes and Israel administrative districts. Abbreviations: C – Central District, H – Haifa District, J – Jerusalem District, N – North District, S – South District, T – Tel Aviv District

The annual incidence rates of infection due to *B. melitensis* biotypes 1 and 2 for the Arab sector according to the administrative districts are shown in Figure 6. It reveals the dynamic changes within the two major *B. melitensis* biotypes across the various administrative districts and the study years. Particularly, a decrease in the annual incidence rate of biotype 1 and an increase in the annual incidence rate of biotype 2 in the South District with opposite trends in the North and Haifa districts were observed. Also, an increase in the annual incidence rate of both biotypes 1 and 2 in Jerusalem District was noted. Comparison of the average incidence rates for the period 2004–2012 versus 2013–2022 (Figure 7) further corroborated the redistribution of *B. melitensis* biotypes 1 and 2 between the southern and northern districts. In the North District, the ratio between the average annual incidence rates of *B. melitensis* biotypes 2 compared to *B. melitensis* biotypes 1 decreased from an IRR of 2.92 (95% CI: 1.34–7.1) in 2004–2012 to an IRR of 0.21 (95% CI: 0.09–0.41) in 2013–2022 (p < 0.01). In the South District, this trend reversed. The average annual incidence rate of *B. melitensis* biotypes 2 compared to *B. melitensis* biotypes 1 increased from an IRR of 0.02 (95% CI: 0.00–0.08) in 2004–2012 to an IRR of 0.49 (95% CI: 0.29–0.8) in 2013–2022 (p < 0.01). The trends in the other districts did not reach statistical significance.

## Discussion

This large-scale national study, based on culture-confirmed *Brucella* infection, underscores the predominance of *B. melitensis* (99.6%), particularly biotype 1 (69.1%) as the causative pathogen in Israel, and highlights the distribution and dynamic changes of *B. melitensis* biotypes over the study years and across geographic locations. The study allows deeper insights into the impact of brucellosis on the various sectors and communities in Israel and reveals the differential epidemiology between the sectors. The study portrays the high level of endemicity and the heavy burden of disease among the Arab sector, the occurrence of periodic large outbreaks among the Druze sector against a background of low incidence rates, and the persistently low incidence rates among the Jewish sector.

This differential epidemiology can be explained in part by animal farming practices. The Arab and Druze sectors more commonly practice traditional mixed sedentary and pastoral farming systems involving family-owned sheep and goat herds, while the Jewish sector more commonly practices commercial farming, which allows better regulation and control [22]. Existing deep-rooted trade connections between Bedouin communities in southern Israel and other regions such as East Jerusalem and the Palestinian Authority, together with door-to-door trade of dairy products among Arab communities, may also contribute to the disparities in the incidence of brucellosis [22, 23]. These and other yet undescribed contributory factors to the different patterns of brucellosis trends require further research.

Culture-confirmed *Brucella* cases were responsible for 50–78% of reported brucellosis diagnoses in recent studies from Israel [23–25]. In concurrence with these results, the average proportion of culture-confirmed cases in our study was 59.5% of the nationally reported cases. Culture-based incidence is considered far more reliable compared to serology-based incidence, since in endemic areas, serology may represent past or recent infections and may lack sensitivity [24]. Serology may also involve a lower positive predictive value among the Jewish sector due to the low incidence of brucellosis in this sector.

Similar to other studies, we found higher incidence rates in males and with increasing age. This may reflect differences in the acquisition route. In certain communities, males are more likely to be in close contact with infected animals due to their role as herd shepherds, while women are more likely to be responsible for milking the animals and preparing traditional dairy products. Such practices could result in different exposure risks for brucellosis [26]. The increase in incidence among males noted in the second decade of life in our study may thus be explained by more intensive exposure to animals, coinciding with the age groups that assume a shepherd role [27].

The seasonal pattern, with higher rates in the spring and summer, is also characteristic [4, 26]. In the Middle East, the seasonal trend is attributed to the parturition season of sheep and goats [26]. High quantities of nutritionally unique milk are obtained during spring grazing and are used to produce traditional dairy products, which are consumed and sold unpasteurized [28].

Our study underlined the persistent endemicity of brucellosis among the Arab sector in Israel. Most of the disease burden was attributed to the Bedouin Arab communities, which are concentrated mainly in southern Israel and in several settlements in northern Israel [12, 13]. Accordingly, the highest incidence rates in our study were found in the South District, reaching a peak incidence rate of 41.0/100,000 population in 2012. The endemicity among the Bedouins is multifactorial. It is related to the traditional raising of sheep and goat herds in close proximity to the family residence, resulting in close contact with the animals and their infected aborted placentas. Brucellosis is endemic in many of these herds due to a lack of efficient control, illegal trade, and frequent animal trafficking across borders from the Palestinian Authority that also suffers a high level of endemicity [17, 22]. Indeed, according to the State Comptroller and Ombudsman of Israel, prior to 2014 about 70% of the herds in southern Israel were not vaccinated due to insufficient veterinary control activities [17]. Ineffective control efforts can also be explained by inadequate compensation schemes for the culled herds and deep mistrust in the authorities. Beyond the financial loss, there is a deeper implication of losing animals that are the main source of income and an important component of family cohesion and solidarity. Family-owned herds also represent the family status in the community and are used for legal claims on pasture lands [29]. Except for direct animal contact, transmission of brucellosis to humans is mostly due to the traditional preference of soft dairy products made from fresh unpasteurized milk, which is not always affected by education about brucellosis and health promotion efforts [30, 31]. In addition to local consumption, childhood brucellosis in East Jerusalem was traced to unpasteurized dairy products purchased in the Palestinian Authority [23].

The annual trends in our study reflect the inconsistency of the brucellosis intervention programmes in Israel [17, 22]. Due to various administrative challenges, national interventions for brucellosis prevention in Israel were mostly short-lived and prematurely terminated before full control was achieved. Following the sharp increase in *Brucella* incidence in the South District, a large-scale intervention programme based on ‘test and slaughter’ and compensation to the herd owners was launched in 2015. The intervention was prematurely stopped in 2017 due to staff shortages and discontinuation of funding [17]. In contrast, the smaller-scale multidisciplinary intervention in Druze settlements such as Yirka was successful [14]. Characteristically, these interventions include more intensive testing and slaughtering and public education through the school system, community centres, and the local media (radio, newspapers, television).

The study portrayed the unique epidemiology of *B. melitensis* biotypes involved in clinical illness in Israel. *B. melitensis* biotype 1 was the dominant serotype responsible for 69.1% of the cases, followed by *B. melitensis* biotype 2 (26.0%) and *B. melitensis* biotype 3 (4.3%). In the Middle East, Eurasia, China, and France, *B. melitensis* biotype 3 is predominant among human isolates, while *B. melitensis* biotype 2 is rarely reported [32–35]. *B. melitensis* biotype 1 was reported as the predominant human biotype in Latin America and Spain and the predominant veterinary biotype in South Africa [36–38]. Our study revealed a redistribution of the *B. melitensis* biotypes over the course of the study years between the southern and northern districts of Israel: *B. melitensis* biotype 2 became more dominant relative to biotype 1 in southern Israel, while opposite trends were observed in northern Israel. A possible explanation for the biotype switch in Israel is the illegal trade of infected animals. In fear of the large intervention planned in the South District, herd owners tended to sell the infected animals outside the district, as has been vividly described in interviews with community representatives and members of the brucellosis intervention target population in southern Israel [29]. While to the best of our knowledge, a substantial proportion of illegal trade involves the Palestinian Authority, the trade of sick animals within Israel may also account for the trends found in our study. Indeed, a recent study exploring the genomic epidemiology of bovine *B. melitensis* in dairy farms identified cases of cryptic transmissions between apparently unrelated farms, suggesting certain veterinary brucellosis transmission routes across the country are yet to be discovered [39]. This finding may be useful to guide future intervention and control efforts.

The strength of our study is being of national scale and based solely on culture-confirmed cases, covering two decades. It provides unique national information on the differential incidence rates among the various sectors and on the distribution of *Brucella* species and biotypes across geographical locations and over the years. This allows a better understanding of the specific components of national trends. Our study did not include serologically confirmed cases, nor epidemiologically linked cases lacking laboratory confirmation, and thus does not reflect the complete incidence rates of brucellosis in Israel. Also, the under-reporting rate of positive cultures is unknown, although it is assumed to be very low since *Brucella* is defined as a select agent per Israeli law and clinical laboratories are prohibited from performing further analysis and referral to the reference laboratory (KVI) is mandatory. Another limitation is that our database could not ascertain local or regional outbreaks of brucellosis although such outbreaks have been shown to occur [14, 23, 40].

While biotyping has been traditionally used for surveillance of brucellosis, its resolution as a typing method is somewhat limited. We have recently shown that human isolates of *B. melitensis* recovered from Bedouin Arab patients in southern Israel involve two main genomic clades, each of which comprises multiple clones. While the predominant biotypes 1 and 2 were grossly segregated into those two clades, intermixing was also evident [40]. Since genomic typing is expensive and requires an appropriate infrastructure, it is not affordable in most *Brucella*-endemic countries. Therefore, biotyping is still being used in resource-limited settings. Biotyping cannot yet be accurately predicted from genomic sequences; therefore, genomic surveillance is important to assess cross-border transmission. In our study, biotyping usefully demonstrated the dynamics of brucellosis over the years, which may coincide with prior reports of illegal trade and animal trafficking. Such information may prove valuable for planning future interventions.

In summary, our study highlights the heavy burden of brucellosis in the Arab and Druze sectors and for the first time reveals the differential epidemiology of these sectors. The study underscores the unique pattern of *B. melitensis* biotypes in Israel, with the predominance of biotype 1. It outlines the dynamics of *B. melitensis* biotypes along the two recent decades, resulting in the redistribution of *B. melitensis* biotypes 1 and 2 between southern and northern Israel. The high endemicity among the Arab and Druze sectors and the findings suggestive of uncontrolled trafficking of affected farm animals call for improved *Brucella* control measures. Being a notable One Health challenge, intervention plans should be sustainable and involve cross-sectoral collaboration involving all major stakeholders, while incorporating the lessons learned from previous intervention efforts [22].

## Data Availability

The data that support the findings of this study are available on request from the corresponding author. The data are not publicly available due to their containing information that could violate the Kimron Veterinary Institute confidentiality requirements.
